# Prawn Shell Chitosan Exhibits Anti-Obesogenic Potential through Alterations to Appetite, Affecting Feeding Behaviour and Satiety Signals *In Vivo*

**DOI:** 10.1371/journal.pone.0149820

**Published:** 2016-02-22

**Authors:** Áine M. Egan, John V. O’Doherty, Stafford Vigors, Torres Sweeney

**Affiliations:** 1 School of Agriculture and Food Science, University College Dublin, Belfield, Dublin, Ireland; 2 School of Veterinary Medicine, University College Dublin, Belfield, Dublin, Ireland; University Paris South, FRANCE

## Abstract

The crustacean shells-derived polysaccharide chitosan has received much attention for its anti-obesity potential. Dietary supplementation of chitosan has been linked with reductions in feed intake, suggesting a potential link between chitosan and appetite control. Hence the objective of this experiment was to investigate the appetite suppressing potential of prawn shell derived chitosan in a pig model. Pigs (70 ± 0.90 kg, 125 days of age, SD 2.0) were fed either T1) basal diet or T2) basal diet plus 1000 ppm chitosan (*n* = 20 gilts per group) for 63 days. The parameter categories which were assessed included performance, feeding behaviour, serum leptin concentrations and expression of genes influencing feeding behaviour in the small intestine, hypothalamus and adipose tissue. Pigs offered chitosan visited the feeder less times per day (P<0.001), had lower intake per visit (P<0.001), spent less time eating per day (P<0.001), had a lower eating rate (P<0.01) and had reduced feed intake and final body weight (P< 0.001) compared to animals offered the basal diet. There was a treatment (P<0.05) and time effect (P<0.05) on serum leptin concentrations in animals offered the chitosan diet compared to animals offered the basal diet. Pigs receiving dietary chitosan had an up-regulation in gene expression of growth hormone receptor (P<0.05), Peroxisome proliferator activated receptor gamma (P<0.01), neuromedin B (P<0.05), neuropeptide Y receptor 5 (P<0.05) in hypothalamic nuclei and neuropeptide Y (P<0.05) in the jejunum. Animals consuming chitosan had increased leptin expression in adipose tissue compared to pigs offered the basal diet (P<0.05). In conclusion, these data support the hypothesis that dietary prawn shell chitosan exhibits anti-obesogenic potential through alterations to appetite, and feeding behaviour affecting satiety signals *in vivo*.

## Introduction

Obesity and obesity-related disorders are reaching epidemic proportions worldwide. The potential of natural products to prevent obesity have been highlighted [1]. There is increasing interest in the use of natural resources as protective agents against obesity because of some harmful side-effects of synthetic compounds [2] Chitosan is a non-toxic nutritional supplement generally regarded as a safe compound [3]. Chitosan is a natural polysaccharide, comprising copolymers of glucosamine (β (1–4)-linked 2-amino-2-deoxy-D-glucose) and N-acetyl glucosamine, (2-acetamido-2-deoxy-D-glucose) which can be derived by partial deacetylation of chitin [4]. Recently chitosan has been shown to improve growth performance in young animals [5]. In contrast chitosan has also received much attention for its anti-obesity potential [6, 7], with dietary supplementation reducing feed intake in both mice [8] and pigs [7]. Ingestion of this natural polysaccharide increased serum leptin, a hormone which plays a key role in appetite suppression, suggesting a potential link between chitosan and appetite control [9].

Feeding behaviour influences energy intake and is itself influenced by appetite-related neuropeptides produced in the central hypothalamus. Within the hypothalamus several nuclei exist which integrate peripheral signals. The main neural pathways involved in appetite control are the orexigenic neuropeptide Y/Agouti-related protein (NPY/AgRp) and the anorexigenic pro-opiomelanocortin/cocaine- and amphetamine-related transcript (POMC/CART) neurons. Signals within these pathways modulate food intake and energy expenditure [10], subsequently regulating pathways within the central nervous system including signals from the gastrointestinal tract (GIT) [11]. The GIT synthesises appetite inducing hormones which are suppressed following food consumption and appetite suppressing hormones which are increased in circulation following food consumption. These include hormones such as peptide YY (PYY), glucagon-like peptide 1 (GLP-1) and cholecystokinin (CCK), ghrelin, insulin, melanocortin and orexin. These hormones act on the hypothalamus, inducing sensations of either hunger or satiety [12]. Meal anticipation and the presence of food in the upper gastrointestinal tract stimulate the release of both gut hormones and neurotransmitters [13]. These signals are involved in the initiation and maintenance of food intake as well as the termination of meals. This system of negative feedback within the gut-brain axis is crucial to appetite regulation and subsequent feeding behaviour [14].

Accumulating evidence suggests that the communication pathways linking the brain, gut and adipose tissue might be promising intervention points for metabolic disorders [15]. The potential of dietary chitosan to modulate the gut-brain-adipose tissue axis in favour of reduced appetite may be contributing to chitosans ability to reduce food intake and body weight gain *in vivo*. Hence, the objective of the present study was to investigate the effect of dietary supplementation of chitosan on feeding behaviour, appetite regulation in the hypothalamus, adipose tissue and intestine, feed intake, body weight gain and serum leptin concentrations. The hypothesis being tested is that dietary supplementation of chitosan would decrease appetite through modulation of hypothalamic, intestinal and adipose tissue regulators of appetite thus reducing feed intake and body weight gain in the pig.

## Materials and Methods

All experimental procedures described in this experiment were conducted under experimental license from the Irish Department of Health in accordance with the Cruelty to Animals Act 1876 and the European Communities (Amendments of the Cruelty to Animals Act, 1876) Regulations (1994). An experimental license (AREC-09-31-O’Doherty) was obtained from the Animal Research Ethics Subcommittee University College Dublin.

### Generation of chitosan from chitin

The chitosan was generated from prawn (*Nephrops norvegicus*) shell sourced from Spiddal Co. Galway, Ireland. Prawn shell was collected on five different occasions before extraction of chitosan. The prawn shell was heated in boiling sodium chloride (4% NaCl) for 10 minutes and cooled in tap water to remove excess prawn protein material. The shell was washed extensively and freeze-dried. Clean, dry shell was milled, sieved and subsequently demineralised and deproteinised using a BioFlo 110 Modular Bioreactor (New Brunswick Scientific, USA). Following this, HCL (0.25 M) was added to the prawn shell material at a ratio of 1:40 weight/volume to demineralise the shell and remove the minerals from the dry shell. The temperature of the reaction was maintained at 40°C for 6 h. The shell material was subsequently drained, washed until a neutral pH was obtained, then frozen and freeze-dried to obtain a demineralised shell powder. The demineralised shell powder was then deproteinised using 0.25 M NaOH using a shell to solvent ratio of 1:40 w/v at 70°C for 6 h. The chitosan was washed until a neutral pH was reached, then freeze-dried and subsequently milled to obtain chitosan powder.

### Characterisation of chitosan

The molecular weight data for the generated chitosan was analysed using the SEDFIT-MSTAR. The degree of acetylation was determined by analysis of the 1H proton spectrum following the method of [16].

### Animals and management

The experiment was a complete randomised design. Forty females pigs (Large White x Landrace genetic lines, Hermitage, Co. Kilkenny, Ireland), with average body weight of 70 kg (SD = 0.9), (125 days of age, SD = 2.0) were randomly assigned to one of two dietary treatments (20 animals/treatment): (T1) basal diet (control) and (T2) basal diet plus 1g/kg chitosan. Diets were offered ad libitum for 63 days. Female pigs were used because of their higher back fat deposition relative to male pigs [17]. The concentration of chitosan used in the present study was based on previous work by Walsh *et al*. [7]. The diets were provided *ad libitum* in a meal form from single space computerised feeders (Mastleistungsprufung MLP-RAP; Schauer Agrotronic AG, Sursee, Switzerland). Water was available *ad libitum* from nipple drinkers. The diets were formulated to have similar digestible energy (14 MJ/kg) and standardised ileal digestible lysine (8.5 g/kg) contents. All amino acid requirements were met relative to lysine [18]. Detailed ingredient composition and chemical analysis of the diets are presented in Table 1.

**Table 1:** Diet composition and chemical analysis (g/kg, unless otherwise indicated).

Ingredient (g/kg)	T1	T2
Wheat	382.6	382.6
Barley	250.0	250.0
Soya bean meal	170.0	170.0
Maize	150.0	150.0
Soya oil	18.0	18.0
Limestone	12.5	12.5
Salt	5.0	5.0
Monocalcium phosphate	6.6	6.6
Vitamins and minerals premix^a^	2.5	2.5
Lysine HCL	2.3	2.3
L-threonine	0.5	0.5
Chitosan	0	1.0
Analysis (g/kg, unless otherwise stated)		
Dry matter	857.6	856.4
Crude protein (N X 6.25)**	177.9	177.7
Neutral detergent fibre	130.5	130.3
Ash	42.5	42.8
Gross energy (MJ/kg)	15.9	15.7
Lysine^†^	9.2	9.1
Methionine and cysteine^†^	5.5	5.4
Threonine^†^	6.2	6.3
Tryptophan^†^	1.9	2.0
Calcium^†^	9.4	9.4
Phosphorous^†^	5.8	5.7

*T1, basal diet; T2, basal diet plus 1g/kg chitosan.

^a^ The premix provided vitamins and minerals (per kg diet) as follows: 4.2 mg of retinol, 0.07 mg of cholecalciferol, 80 mg of α-tocopherol, 120 mg of copper as copper sulphate, 100 mg iron as ferrous sulphate, 100 mg of zinc as zinc oxide, 0.3 mg of selenium as sodium selenite, 25 mg of manganese as manganous oxide, 0.2 mg of iodine as calcium iodate on a calcium sulphate/calcium carbonate carrier, 2 mg of thiamine, 15 μm of cyanocobalamin, 7 mg of pantothenic acid, 2 mg of riboflavin, 7 mg of niacin, 3 mg of adenine and 100 mg of phytase (Natuphos) (Nutec, Co. Kildare, Ireland).

**Crude protein (Nitrogen X 6.25).

^†^ Calculated for tabulated nutritional composition [20].

The animals were penned in four groups of ten with a space allowance of 0.75m^2^ per pig. The pens were equipped with single space computerised feeders (Mastleistungsprufung MLP-RAP; Schauer Agrotronic AG, Sursee, Switzerland), as described by Varley *et al*. [19] and Walsh *et al*. [7] which allowed individual *ad libitum* feeding and daily recording of dietary intake. Briefly, when the animal entered the feeder, it was recognised by the electronic system (MLP-Manager 1.2; Schauer Maschinenfabrik Ges.m.b.H and CoKG, Prambachkirchen, Austria). Each animal was ear-tagged with a uniquely coded transponder and the identification circuit recorded the number of the animal. When the animal finished feeding and withdrew from the trough, the electronic system recorded the difference between the pre- and post-visit trough weight and the data was stored in a file with the number of identification of the animal, the date, and the time of entry and exit. The animals were weighed at the beginning of the experiment (day 0) and every two weeks up to slaughter (day 63).

### Blood sample collection

Blood samples (10 ml) were collected from each animal (n = 10) from the *vena jugularis* by puncture into vacutainers (Becton, Dickinson, Drogheda, Ireland) on day 0 (prior to commencing of the experiment), day 14, 28, 37, 49 and 63 to facilitate leptin quantification. Blood samples were allowed to clot at 4°C and serum was collected after centrifugation (1,500 × g for 15 min at 4°C). Serum samples were stored at -20°C until analysis.

### Post slaughter sample collection

On day 63, all animals were slaughtered after stunning with an electrical bolt and the entire intestinal tract and brain were removed by blunt dissection. Three regions of the brain were dissected from the hypothalamus to collect; the paraventricular nucleus (PVN), arcuate nucleus (ARC) and the lateral hypothalamic area (LHA) to accommodate gene expression analysis of hypothalamic regulators of appetite and feeding behaviour: Neuropeptide Y (*NPY*), Pro-opiomelanocortin (*POMC*), Agouti related protein (*AgRp*) and Cocaine amphetamine regulated transcript (*CART*), Orexin (*HCRT*) and Neuromedin (*NMB*), Growth hormone receptor (*GHR*), Insulin receptor (*INSR*) and Peroxisome proliferator activated receptor gamma (*PPARG*). Brain tissue samples were stored in RNAlater solution (Ambion Inc, Austin, TX) overnight at 4°C. Tissue samples from the jejunum (60 cm from the stomach) and ileum (10 cm from the ileo-cecal valve) were collected to analyse the gene expression of appetite gut hormones: Cholecystokinin (*CCK*), Peptide YY (*PYY*) Glucagon-like peptide 1 (*GLP-1*) and Neuropeptide Y (*NPY*). Intestinal tissue samples were emptied and cleaned by dissecting along the mesentery and rinsing using sterile PBS (Oxoid) as described previously [21, 22]. Tissue sections of 1 cm^2^, which had been stripped of the overlying smooth muscle were cut from the tissue and stored in RNAlater solution (Ambion Inc, Austin, TX) overnight at 4°C. The RNAlater™ was then removed from brain and intestinal tissue samples and samples were stored at -70°C until RNA extraction. Adipose tissue was collected from the mid-region of the back and snap frozen in liquid nitrogen and transported to the laboratory and stored at -70°C until RNA extraction. Adipose tissue samples were collected to analyse the gene expression of *leptin*.

## Laboratory Analysis

### Leptin quantification—ELISA

Serum leptin was quantified by using a specific pig leptin enzyme-linked immunosorbent assay (ELISA) kit from Life Science Inc. (Wuhan, China) according to the manufacturer’s instructions. Sensitivity of the assay was 0.114 pg/ml, and intra-assay coefficient of variation was < 12%. Absorbance was measured at 450 nm against 570 nm for each assay by using the ELISA plate reader. All samples were assayed in triplicate in the same assay.

### RNA extraction, complementary DNA synthesis and quantitative PCR

Total RNA was purified using the trizol extraction method. Total RNA was extracted from approximately 50 mg of adipose tissue and small intestinal samples and from the entire brain tissue sample using the GenElute™ Mammalian Total RNA Miniprep Kit (Sigma-Aldrich,Corporation) according to the manufacturer's instructions. Total RNA samples were treated with DNase I (Sigma-Aldrich). Total RNA was quantified using a NanoDrop-ND1000 spectrophotometer (Thermo Fisher Scientific, Inc.). RNA integrity was assessed on the Agilent 2100 Bioanalyze version A.02.12 (Agilent Technologies, Inc.) and all RNA integrity number values were > 8.9. Complementary DNA (cDNA) was synthesised from 1 μg of total RNA using the Superscript™ III First-Strand Synthesis Kit (Thermo Scientific) and oligo (dt) primers following the manufacturer's instructions. The final reaction volume of 20 μl was then adjusted to 120 μl using nuclease-free water. The quantitative PCR (RT-qPCR) assay mixtures were prepared in a total volume of 20 μl, containing 10 μl Fast SYBR PCR Master Mix (Applied Biosystems, Foster City, CA, USA), 1.8 μl forward and reverse primer mix (300 nm), 5.7 μl nuclease-free water and 2.5 μl cDNA. The RT-qPCR was carried out in duplicate on the 7500 ABI Prism Sequence Detection System (Applied Biosystems, Foster City, CA, USA). Thermocycling conditions were as follows: 95°C for 10 min for one cycle, followed by 95°C for 15 s and 60°C for 1 min for forty cycles. Dissociation analyses of the QPCR products confirmed the specificity of all targets. All primers for the selected genes: *NPY*, *POMC*, *AgRp*, *CART*, *HCRT*, *NMB*, *GHR*, *PPARG*, *INSR*, *CCK*, *GLP-1*, *PYY* and *leptin* are presented in Table 2. All primers were designed using the Primer Express^TM^ Software (Applied Biosystems, Foster City, CA, USA) and synthesised by MWG Biotech (Milton Keynes, Buckinghamshire, UK). Dissociation analyses of the QPCR products were carried out to confirm the specificity of the resulting QPCR products. All samples were prepared in duplicate. The mean cycle threshold values of duplicates of each sample were used for calculations. The optimal number of reference targets were identified using the geNorm application within the qbase PLUS software package (Biogazelle, Zwijnaarde, Belgium). Briefly, the geNorm algorithm on the qbase+ package (Biogazelle, Gent, Belgium) calculated the expression stability factor (M). From this the optimal combination of reference genes required for normalisation were selected. Using this algorithm, reference genes are ranked based on their M values. In brief, geNorm calculates the stability measure M for a reference gene as the average pairwise variation (V) for that gene with all other tested reference genes. A Vn/n+1 value is calculated for every comparison between two consecutive numbers (n and n+1) of candidate reference genes. Following the stepwise exclusion of the least stable reference genes, by the geNorm program, M values were re-calculated and the stability series obtained. Finally, the NF was calculated, as the geometric mean of the most stable reference genes, and the normalised relative quantity (NRQ) of the target genes obtained as the ratio between the relative quantities and the sample specific NF. The basic formula for relative quantification (RQ = 2^ddCt) assumes 100% amplification efficiency (E = 2). The most stable housekeeping genes selected for the ARC were; Glyceraldehyde 3-phosphate dehydrogenase (*GAPDH*) and hydroxymethylbilane synthase (*HMBS*), PVN; Beta actin (*ACTB*) and Peptidylprolyl isomerase A (*PPIA)*, LHA; hydroxymethylbilane synthase (*HMBS)* and Glyceraldehyde 3-phosphate dehydrogenase (*GAPDH)* and adipose tissue; Peptidylprolyl isomerase A (*PPIA)* and *HMBS*, ileum and jejunum; *PPIA* and *HMBS*.

**Table 2:** Swine-specific primers used for brain, intestinal and adipose tissue real-time PCR.

Gene*	Accession no.	Primer (5' → 3')	Product Length	T_m_ (°C)
*PYY*	XM_005668763.1	F; CTCCTGATTCGGTTTGCAGAA	61	57.9
		R; GGACAGGAGCAGCAGGAAGA		61.4
*CCK*	NM_214237.2	F; GGACCCCAGCCACAGAATAA	61	59.3
		R; GCGCCGGCCAAAATC		56.3
*GLP-1*	NM_001256594.1	F; CAGTGCAGAAATGGCGAGAA		64.3
* *		R; GGTGGAGCCTCAGTCAGGAA	61	62.5
*Leptin*	NM_213840.1	F: AAAGCCTGCCTGTTTGCTCAT		57.9
		R: AGAAAGCGACGGTGAGTTGTG		59.8
*GHR*	NM_214254.2	F: CAGCAGGGAGTGTGGTCCTT	67	61.4
		R: TGCATGTCACACTGGGAGATC		59.8
*NPY*	NM_001256367.1	F: CAGGCAGAGATACGGAAAACG	71	59
		R: TCCGTGCCTCTCTCATCAAG		59.1
*POMC*	NM_213858	F: CCTGGTCACGCTGTTCAAAA	63	57.3
		R:: AACCCTCACTGGCCCTTCTT		59.4
*CART*	NM_001099925.1	F: TACCCCCCCCCAACACA	68	57.6
		R: TGCTAAAGCCAGGGATGAAAG		57.9
*AgRp*	NM_001011693.1	F: CAGAGGTGCTAGATCCTGAAGGA	91	62.4
		R: GACAGGATTCGTGCAGCCTTA		59.8
*NPY5R*	XM_003129011.2	F; GGGCCTTGCCATTTGCT	65	55.2
		R; CAAAGCTTTCCTGGAGTTCCA	68	57.9
*NMB*	EU375564.1	F; AGCATCTCACACCCCGTACAG		61.8
		R; TTCCTGATTCGTGGCATCAC	77	61.8
*HCRT*	NM_214156.2	F; GGCTATTCAGACCACGGAAGAC		62.1
		R; CAAAAGGAGATTCATGGTGTCA	65	58.9
*INSR*	XM_005654749.1	F; TGCATACCTGAACGCCAAGAAG		61.1
		R; GGGCGACCATGCAATTTC	66	57.1
*PPARG*	AF103946.1	F; TGTCTCATAACGCCATCAGGTT		57.3
		R; TCTCTGCCAACAGCTTCTCCTT	71	58.4

F, forward; R, reverse

*PYY, Peptide YY; *CCK*, Cholecystokinin; *GLP-1*, Glucagon like peptide 1; *HMBS*, hydroxymethylbilane; *PPIA*, Peptidylprolyl isomerase A; *NPY*, neuropeptide Y; *POMC*, Pro-opiomelanocortin; *CART*, cocaine amphetamine regulated transcript; *AgRp*, agouti related protein; *NPY5R*, Neuropeptide Y 5 receptor; *NMB*, neuromedin B; *HCRT*, Orexin; *INSR*, insulin receptor; *PPARG*, Peroxisome Proliferator-Activated Receptor Gamma.

### Statistical analysis

The growth performance was analysed by repeated measures analysis using the PROC MIXED procedure of SAS [23]. The model included pen and animal within pen as random effects. The fixed effects were: treatment, time and interaction between treatment and time. The carcass data was analysed using the PROC MIXED procedure of SAS. The model used included pen and animal within pen as random effects. The fixed effect was treatment. The leptin data was analysed by repeated measures analysis using the PROC MIXED procedure of SAS [23]. The model used included the pig as a random effect. The fixed effects were: treatment, time of sampling and the associated two way interaction between treatment and time of sampling. The data on adipose tissue, brain and small intestinal gene expression were analysed using the general linear model procedure of the Statistical Analysis Systems Institute [24]. The model used included the effect of treatment. Feeding behaviour data was analysed by repeated measures analysis using the PROC MIXED procedure of SAS. The model used included the effect of treatment. The probability level that denotes significance is P< 0.05, while P values between 0.05 and 0.1 are considered numerical tendencies. Data are presented as least-square means with their standard errors.

## Results

### Characterisation of chitosan

The degree of acetylation obtained was 15%. The molecular weight of the prawn shell chitosan was 124,000 ± 10,000 g/mol.

### Feed intake and body weight

**Fig 1:**
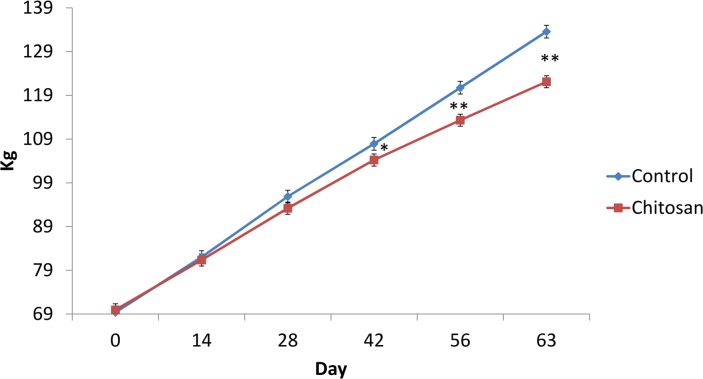
Effect of dietary supplementation on body weight over time at days 0, 14, 28, 42, 56 and 63. *P<0.05 **P<0.001. Treatment effect P< 0.001. Time effect P< 0.001. Time x treatment effect P< 0.01. Values are means, with their standard errors represented by vertical bars.

**Fig 2:**
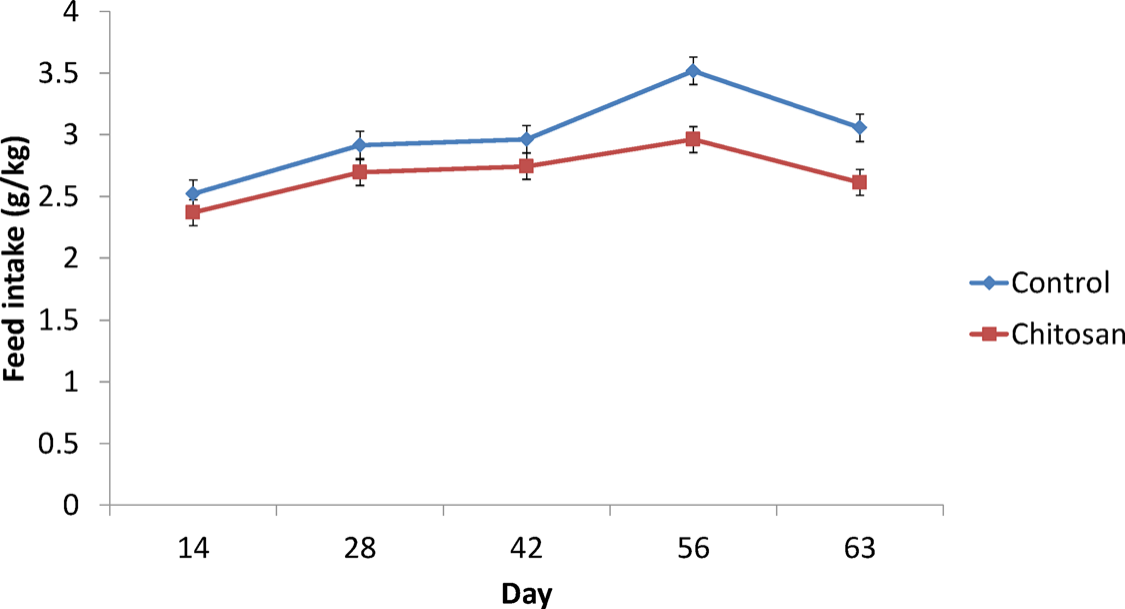
Effect of dietary supplementation on feed intake over time at days 0, 14, 28, 42, 56 and 63. **Treatment effect P<0.001. Time effect P<0.001.** Values are means, with their standard errors represented by vertical bars.

The effect of chitosan supplementation on body weight and feed intake over time are presented in Figs 1 and 2 while the effect of chitosan supplementation on pig performance and carcass characteristics are presented in Table 3. There was a time x treatment interaction (P< 0.01) on body weight, where chitosan supplemented pigs had a lower body weight on day 56 (P<0.01) and day 63 (P<0.001) compared to the control group. There was a treatment effect (P< 0.001) and time effect (P< 0.001) on feed intake. Pigs offered the chitosan diet had lower feed intake (*P*< 0.01) and body weight gain (*P*< 0.05) during the experiment (days 0–63) (*P*< 0.01), and lower final body weight (*P*< 0.05) compared with basal diet fed pigs. Pigs receiving chitosan had lower carcass fat content compared to basal diet fed pigs (P< 0.05).

**Table 3:** Effect of dietary supplementation on growth performance and carcass characteristics (least-square means and SEM).

Performance	Control	Chitosan	SEM	Significance
Starting BW (kg)^†^	70.0	70.0	0.900	1.00
Feed intake (kg/d)	2.99	2.67	0.05	0.001
Body weight gain (kg/d)	0.88	0.79	0.03	0.042
Feed efficiency ratio (kg/kg) ^††^	3.57	3.30	0.37	0.593
Final BW(kg)^†^	125.6	119.3	1.87	0.001
Carcass fat content (kg) ^†††^	36.4	33.6	0.74	0.012

BW, body weight; SEM, standard error of mean.

^†^ Starting BW = day 0; final BW = day 63.

^††^ Body weight gain/ feed intake.

^†††^Carcass fat content = carcass weight–(lean + ash content of carcass).

### Feeding behaviour

The effect of dietary supplementation of chitosan on feeding behaviour is shown in Table 4. Pigs offered the chitosan diet ate less per visit (*P* <0.001), had a lower number of visits per day (*P* <0.001), spent less time eating per day (*P* <0.001) and had a lower eating rate (*P* <0.01) when compared to the control group.

**Table 4:** Effect of prawn shell chitosan on feeding behaviour (D0-63) (least square means and SEM).

Behaviour	Control	Chitosan	SEM	Significance
Number visits/day	20.304	15.819	0.2555	0.0010
Intake/visit (g)	182.89	198.48	2.5396	0.0010
Total time eating/day (Seconds)	5975.55	5438.56	80.115	0.0010
Eating rate (g/second)	0.4694	0.4478	0.0048	0.0018

### Serum leptin

**Fig 3:**
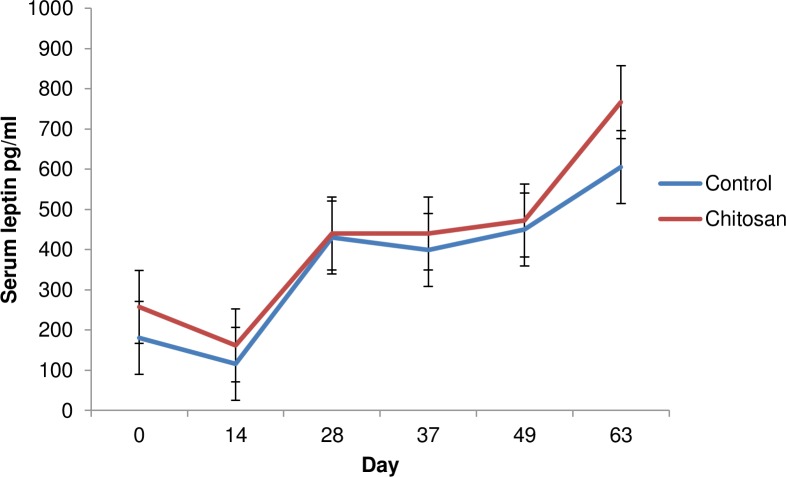
Effect of dietary supplementation on serum leptin levels over time at days 0, 14, 28, 37, 49 and 63. Treatment effect (P<0.05). Time effect (P<0.05). Values are means, with their standard errors represented by vertical bars.

There was a time effect (*P*<0.05) and treatment effect (*P*<0.05) on serum leptin concentrations. Serum leptin concentrations were increased in animals offered the chitosan diet compared with the control group (*P*< 0.05) (Fig 3).

### Hypothalamic regulators of appetite

The effects of dietary supplementation of chitosan on hypothalamic regulators of appetite are shown in Table 5. Dietary chitosan up-regulated *GHR* gene expression (*P* <0.05) and down-regulated *HCRT* expression in the ARC (P<0.05) when compared to the control group. Dietary chitosan resulted in an up-regulation of *PPARG* (*P*<0.01) *NMB* and *NPY5R* (*P*<0.05) gene expression in the PVN when compared to the control animals. Dietary chitosan had a tendency to up-regulate *INSR* (*P* = 0.07) in the PVN when compared to the control group. There was no effect of diet on hypothalamic regulators of appetite in the LHA (P>0.05).

**Table 5:** Effect of dietary treatment on hypothalamic regulators of appetite in the ARC, PVN and LHA (least square means and SEM).

Treatment	Control	Chitosan	SEM	Significance
Arcuate nucleus (ARC)				
NPY	1.470	1.481	0.3140	0.980
POMC	1.550	1.114	0.2965	0.314
CART	1.454	1.570	0.4699	0.864
AgRp	2.301	2.041	0.5800	0.755
PPARG	1.005	1.131	0.1671	0.603
INSR	1.016	1.027	0.1215	0.952
GHR	0.670	1.721	0.3070	0.034
HCRT	2.223	0.811	0.3733	0.021
NMB	1.021	1.015	0.1753	0.981
NYP5R	0.913	1.184	0.1250	0.154
Paraventricular nucleus (PVN)				
NPY	1.125	0.952	0.3528	0.734
POMC	0.865	0.882	0.1521	0.939
CART	0.672	1.233	0.5520	0.174
AgRp	0.777	0.620	0.0907	0.243
PPARG	0.448	1.263	0.1761	0.007
INSR	0.645	2.461	0.6495	0.079
GHR	0.927	1.695	0.5411	0.346
HCRT	1.492	1.985	0.9721	0.730
NMB	0.772	1.582	0.3012	0.050
NYP5R	0.275	2.288	0.6133	0.045
Lateral hypothalamic area (LHA)				
NPY	1.543	0.992	0.3786	0.319
POMC	1.087	1.216	0.2141	0.675
CART	1.278	1.938	0.5608	0.418
AgRp	1.060	1.281	0.2361	0.518
PPARG	1.206	1.750	0.3569	0.329
INSR	0.938	1.668	0.3036	0.113
GHR	1.870	2.334	0.9367	0.731
HCRT	4.190	4.392	1.2844	0.923
NMB	1.463	1.807	0.4457	0.614
NYP5R	1.172	1.422	0.3210	0.591

*NPY*, Neuropeptide Y; *POMC*, Pro-opiomelanocortin; *CART*, cocaine amphetamine regulated transcript: *AgRp*, Agouti related protein: *PPARG*, Peroxisome proliferator activated receptor gamma; *INSR*, insulin receptor; *GHR*, Growth hormone receptor: *HCRT*, Orexin; *NMB*, Neuromedin B: *NPY5R*, Neuropeptide Y 5 receptor: SEM, standard error of mean.

### Gene expression of gut and adipose tissue appetite hormones

The effect of chitosan supplementation on small intestinal appetite gene expression is presented in Table 6. Dietary supplementation of chitosan down-regulated *NPY* gene expression in the jejunum when compared to the control group (*P*<0.05). There was no effect of dietary supplementation of chitosan on the remaining genes analysed (*P* >0.05). Animals offered the chitosan diet had an up-regulation of *leptin* gene expression in adipose tissue compared to the control group (P<0.05).

**Table 6:** Effect of dietary treatment on small intestine and adipose tissue regulators of appetite gene expression (least square means and SEM).

Treatment	Control	Chitosan	SEM	Significance
Jejunum				
*PYY*	1.442	1.087	0.3469	0.4922
*CCK*	1.110	1.092	0.1724	0.9439
*GLP-1*	1.164	0.993	0.1670	0.4835
*NPY*	2.767	0.880	0.5048	0.0262
Ileum				
*PYY*	0.914	1.386	0.4065	0.4445
*CCK*	0.724	1.136	0.1879	0.1675
*GLP-1*	1.145	1.092	0.3662	0.9224
*NPY*	1.377	1.070	0.4702	0.6637
Adipose tissue				
*Leptin*	0.963	1.445	0.1679	0.0500

*PYY*, Peptide YY; *CCK*, Cholecystokinin; *GLP-1*, Glucagon like peptide 1; *NPY*, Neuropeptide Y; Leptin, SEM, standard error of mean.

## Discussion

The polysaccharide chitosan has anti-obesogenic effects *in vivo* [6, 7]; however the anti-obesogenic mode of action of chitosan is poorly understood. The present study hypothesised that dietary supplementation of chitosan would decrease appetite through modulation of hypothalamic, intestinal and adipose tissue regulators of appetite, thus reducing feed intake and body weight gain in the pig. The responses observed in animals offered chitosan support this hypothesis. These responses included down-regulation of orexigenic genes in the small intestine and hypothalamus, reduced feed intake and weight gain, increased serum leptin and up-regulated leptin expression in adipose tissue.

In order to maintain body weight, the brain must tightly monitor the peripheral energy state. This is governed by genes influencing appetite and feeding behaviour. Two major groups of metabolic inputs inform the brain about the peripheral energy state: short-term signals produced by the gut system and long-term signals produced by adipose tissue. After central integration of these inputs, the brain generates neuronal and hormonal outputs to balance energy intake with expenditure. The brain continually monitors the systemic metabolic state and adjusts behaviour, as well as humoral and neuronal outputs to peripheral organs, to maintain body weight and avoid excess weight loss or gain. The potential of dietary chitosan to modulate the gut-brain-adipose tissue axis in favour of reduced appetite may be contributing to chitosans ability to reduce food intake and body weight gain *in vivo*. Information about energy stores and recent food intake is communicated between the hypothalamus and intestine ultimately influencing the perception of hunger, satiety and satiation [25]. In the present study, chitosan supplemented animals had down regulated *NPY* gene expression in the jejunum. Neuropeptide Y is found in both the brain and nervous system [26]. With regard to appetite stimulation, NPY is a potent peptide which increases food intake [27, 28]. The down-regulation of *NPY* gene expression in the jejunum of chitosan supplemented animals may have contributed to the decrease in feed intake observed in the present study. This may be explained by the fact that the hypothalamus receives neural and endocrine signals from the gut in response to food intake. These signals are integrated, interpreted and directed to other centres in the brain and peripheral organs to orchestrate energy homeostasis. Furthermore, analysis of hypothalamic gene expression identified that chitosan supplemented animals exhibited a down-regulation of Orexin/HCRT gene expression in the arcuate nucleus. The hypothalamic neuropeptide orexin regulated appetite through successful stimulation of food intake in weanling pigs [29]. The down-regulation of *orexin* gene expression in the ARC in the present study may have resulted in the chitosan supplemented animals having altered feeding behaviour. Animals receiving dietary chitosan had reduced feed intake; subsequently this group of animals had reduced body weight gain. Reduced dietary energy consumption is positively related to reduction in body weight gain [30]. Additionally, chitosan supplemented animals expressed more *NMB* (Neuromedin B) in the paraventricular nucleus. A member of the bombesin family, NMB reduced food intake when administered systemically in humans and animals [31, 32]. Neuromedin B has a role in the short-term control of food intake, reducing the quantity of food consumed at any given meal and increasing both satiety and satiation. This was evident in this study as supplementation with chitosan resulted in the animals engaging in less visits to the feeder, consuming less feed per visit while also reducing the feeding rate. When administered between meals, NMB increases the amount of time between meals [33, 34]. Furthermore, in the present study we observed an up-regulation of *GHR* gene expression in the arcuate nucleus of chitosan supplemented animals while *NPY5R* was also up-regulated in the paraventricular nucleus. Growth hormone, a protein released into the circulation from the anterior pituitary increases food intake [35]. Similarly, neuropeptide Y, a well characterised potent peptide also increases food intake [36]. Studies have demonstrated that NPY stimulates the release of GH and restores appetite [37, 38]. As NPY and GH are involved in increasing appetite, it may be postulated that the gene expressions of both receptors were up-regulated in an attempt to restore appetite. However, restoration of appetite did not appear to ensue as chitosan supplemented animals had lower feed intake when compared to the control animals. Although indeterminate, these findings may be attributed to negative feedback loops; whereby receptors sense changes in function and initiate the body's homeostatic response [39]. For instance, when adiposity levels increase or decrease, the brain triggers physiological homoeostatic mechanisms which resist weight change through compensatory changes in appetite [40, 41].

Obesity is frequently associated with diabetes mellitus, a group of metabolic diseases characterised by hyperglycaemia resulting from defects in insulin secretion, insulin action, or both. Insulin receptors are widely expressed in the brain, particularly in hypothalamic nuclei, which are involved in control of food intake [42]. The main activity of the insulin receptor is the induction of glucose uptake. Insulin insensitivity, or decreases in insulin receptor signalling, leads to diabetes mellitus type 2. In the present study, chitosan supplemented animals had a tendency for up-regulated *INSR* gene expression in the paraventricular nucleus. This observation suggests that dietary chitosan may have the potential to improve insulin sensitivity *in vivo* and is consistent with previous studies which have demonstrated that chitosan increases glucose tolerance and insulin secretion making it a potential antidiabetic agent [43, 44]. Similar to this, in the present study *PPARγ* expression was up-regulated in the paraventricular nucleus of chitosan supplemented animals. The PPAR activation in type 2 diabetic patients results in a marked improvement in insulin and glucose parameters, resulting from an improvement of whole-body insulin sensitivity [45]. Peroxisome proliferator-activated receptor gamma is a nuclear receptor that regulates an array of diverse functions such as growth and differentiation in a variety of cell types [46].While *PPARγ* is predominantly expressed in adipose tissue it is also expressed/present in neurons [47]. Disruption of PPARγ predisposes mice to the development of diet-induced obesity, insulin resistance, and glucose intolerance [48], whereas activation of PPARγ within macrophages promotes lipid efflux [49]. Furthermore, while PPARγ is associated with adipogenesis [46], chitosan supplemented animals had a lower carcass fat content when compared to the control group. This is an interesting observation as studies have suggested that the up-regulation of PPARγ could potentially up-regulate lipid efflux [50]. This is an interesting theory as it has been generally accepted that the anti-obesity effects of chitosan originate from its unique fat-binding properties, which interferes with the absorption of dietary lipids from the gastrointestinal tract [51, 52].

Energy homeostasis is governed by a complex neuroendocrine system including adipocyte-derived peripheral signals such as leptin [53]. Leptin informs the brain about whole-body long-term energy-storage status, and adipose tissue drives the brains control of energy balance and the long-term regulation of body weight [15]. The ability of leptin to regulate appetite and energy expenditure in rodents with subsequent loss of adipose tissue has led to leptin being termed the anti-obesity hormone [54]. In the present experiment, leptin gene expression was up-regulated in the adipose tissue of the chitosan supplemented animals. Furthermore serum leptin concentrations were higher in chitosan supplemented animals. This rise in the circulation of leptin may have communicated feelings of increased satiety to the brain of animals receiving dietary chitosan. Leptin administration to both wild-type and ob/ob mice reduces food intake and body weight significantly [55]. While the rate of leptin production is related to adiposity [56], it appears dietary chitosan had a direct effect on both leptin production and circulation, as leptin concentration appeared to be independent of level of adiposity. In support of this, chitosan supplemented animals had a lower body weight and carcass fat content compared to the control animals. Furthermore, it has been reported that exposure of human adipose tissue to glucosamine increased leptin release in culture medium [57]. The findings of the aforementioned study demonstrate that adipocytes (isolated from morbidly obese subjects) significantly increase leptin release in the presence of glucosamine. Similarly, the addition of glucosamine to adipocytes from lean subjects also resulted in a significant increase in leptin release [57]. This is interesting as chitin and chitosan are polymers of N-acetyl glucosamine and glucosamine units, respectively [58].

Chitosan has been shown to exert minimal to significant anti-obesogenic effects [59, 60]. The likely rationale behind these variable results is unclear and may be related to body weight of the animals at supplementation. Interestingly, dietary chitosan reduced feed intake in pigs after 100kg body weight was attained, while no response was observed in pigs weighing less than 100kg [7]. Previously, appetite of animals was found to be suppressed only by feeding an excessive amount of chitooligosaccharide (COS) for a long term (189 days) [61]. Interestingly, the present study using medium molecular weight chitosan (124,000 ± 10,000 g/mol) identified reductions in feed intake throughout the entire experiment (63 days) where chitosan was included at a rate of 1.0g/kg. The biological effects of chitosan are highly dependent on its molecular weight, solubility and deacetylation percentage, with low molecular weight chitosan readily absorbed by intestinal cells in vitro and in vivo [62, 63].

In summary, while chitosan has been shown to exert anti-obesogenic potential (Walsh et al., 2013), the present study identified novel mechanisms through which chitosan alters appetite and feeding behaviour in a pig model. These mechanisms included alterations to genes influencing both appetite and feeding behaviour within the brain (*HCRT*, *INSR*, *NMB*, *GHR*, *PPARG*, *NPY5R*), small intestine (*NPY*) and adipose tissue (*Leptin*). This is subsequently followed by the animals exhibiting altered feeding behaviour. The alterations to feeding behaviour show evidence of reduced appetite as the pigs visit the feeders less per day and subsequently consume less feed. This reduction in daily consumption then leads to reduced body weight gain thus lower body weights. The findings of this study suggest that chitosan may be an effective anti-obesogenic agent for inclusion in the human diet.
